# iRhom1 regulates proteasome activity via PAC1/2 under ER stress

**DOI:** 10.1038/srep11559

**Published:** 2015-06-25

**Authors:** WonJae Lee, YoungDoo Kim, Jisu Park, SangMi Shim, Jieun Lee, Se-hoon Hong, Hye-Hyun Ahn, Huikyong Lee, Yong-Keun Jung

**Affiliations:** 1 Global Research Laboratory, School of Biological Science/N-bio Institute, Seoul National University, 1 Gwanak-ro, Gwanak-gu, Seoul 151-747, Korea

## Abstract

Proteasome is a protein degradation complex that plays a major role in maintaining cellular homeostasis. Despite extensive efforts to identify protein substrates that are degraded through ubiquitination, the regulation of proteasome activity itself under diverse signals is poorly understood. In this study, we have isolated iRhom1 as a stimulator of proteasome activity from genome-wide functional screening using cDNA expression and an unstable GFP-degron. Downregulation of iRhom1 reduced enzymatic activity of proteasome complexes and overexpression of iRhom1 enhanced it. Native-gel and fractionation analyses revealed that knockdown of iRhom1 expression impaired the assembly of the proteasome complexes. The expression of iRhom1 was increased by endoplasmic reticulum (ER) stressors, such as thapsigargin and tunicamycin, leading to the enhancement of proteasome activity, especially in ER-containing microsomes. iRhom1 interacted with the 20S proteasome assembly chaperones PAC1 and PAC2, affecting their protein stability. Moreover, knockdown of iRhom1 expression impaired the dimerization of PAC1 and PAC2 under ER stress. In addition, iRhom1 deficiency in *D. melanogaster* accelerated the rough-eye phenotype of mutant Huntingtin, while transgenic flies expressing either human iRhom1 or *Drosophila* iRhom showed rescue of the rough-eye phenotype. Together, these results identify a novel regulator of proteasome activity, iRhom1, which functions via PAC1/2 under ER stress.

The ubiquitin-proteasome system (UPS) is one of the primary clearance machineries that participate in the degradation of regulated, malfunctioned, misfolded, and damaged proteins by marking them with a poly-ubiquitin chain for loading onto the 26S proteasome^1^,^2^. This elaborate clearance occurs in various cellular compartments, including the nucleus, mitochondria, and endoplasmic reticulum (ER)^3^,^4^,^5^. For example, the UPS is responsible for degradation of Mfn1 and Mfn2 mitochondrial fusion proteins in the cytosol^6^ and degrades nuclear FANC2, ATM, and ATR proteins in response to DNA damage signals in the nucleus^7^. In the ER, many secretory and transmembrane proteins are folded during synthesis and checked for the correct folding by this protein quality control system^8^. Misfolded proteins are eventually retro-translocated into the cytosol by ER-associated proteins for degradation by the UPS, an ER-associated degradation (ERAD) process^9^.

Increasing evidence has shown that the activity and assembly of the proteasome are regulated by specific signals. During IFN-γ signaling, for example, the immunoproteasome is assembled by the induction of several immune-associated subunits, such as βi or PA28^10^. It also has been reported that the level of the 20S proteasome assembly chaperone POMP is increased by IFN-γ^11^. TNF-α signaling has been shown to induce S5b/PSMD5, one of the 19S base proteasome assembly chaperones, which inhibits the assembly and activity of the 26S proteasome by recruiting the proteasomal subunit S7^12^. Conversely, deletion of S5b/PSMD5 enhances proteasome activity in *D. melanogaster* and rescues the rough-eye phenotype of the tau fly model. In addition, mild inhibition of the proteasome by proteotoxic stress, such as that induced by the proteasome inhibitor MG132, leads to increased level of TCF11, a major transcription factor for proteasome subunits, and increases the number of proteasomes^13^. The thymus expresses the unique proteasome subunit β5t and produces a thymus-specific proteasome complex that is critical for CD8+ cell development^14^. These previous findings suggest that the proteasome is regulated in a signal- and tissue-specific manner with physiologic and pathologic relevance.

The iRhom1 and 2 are counter parts of drosophila iRhom, member of the Rhomboid protease family that is located in the ER and functions to process EGF or TGF-α. In contrast to other Rhomboid protease family members, iRhom lacks protease catalytic activity and acts as a pseudoprotease that inhibits translocation of EGF ligand family members to the Golgi by binding to them and targeting them to the proteasome. In a *D. melanogaster* model, loss of drosophila iRhom leads to increased sleep periods as a result of the hyperactivation of EGFR signaling^15^. In mammal, iRhom1 and 2 also participate promoting the degradation of EGF^16^. Especially, iRhom2 is essential for TACE trafficking and processing to control TNF in hematopoietic cell^16^,^17^,^18^ and iRhom1 plays a role in survival of several epithelial cancers^19^ and in the suppression of HIF-α degradation in breast cancer cells^20^.

To identify novel factors or signals that regulate proteasome activity, we performed a functional screening and found that iRhom1 regulated proteasome activity independently of EGF signaling. In particular, the expression of iRhom1 was increased under ER stress and thus enhanced proteasome activity, possibly via PAC1 and PAC2.

## Results

### iRhom1 isolated by functional screening enhances proteasome activity

**Figure 1:**
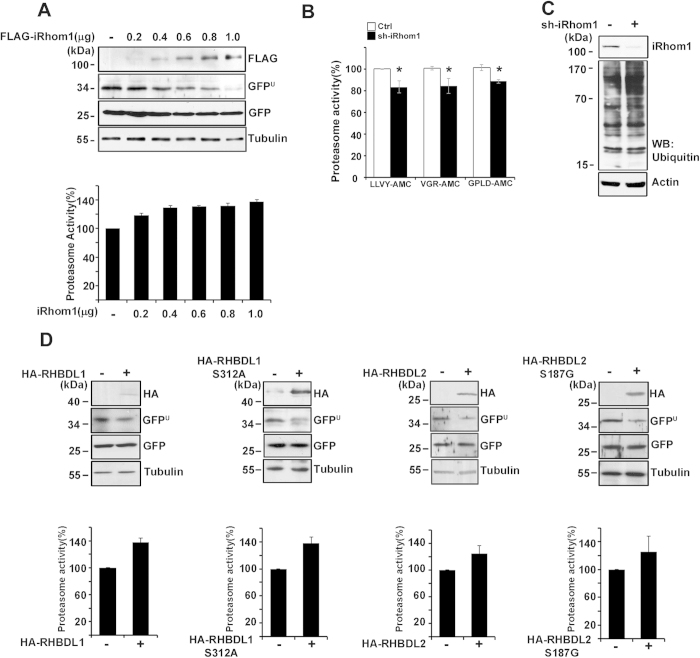
Expression level of iRhom1 affects proteasome activity. (**a**) Ectopic expression of iRhom1 reduces degron (GFP^U^) and elevates proteasome catalytic activity. HEK293T cells were cotransfected with GFP^U^ and the indicated concentrations of FLAG-iRhom1 for 30 h. Cell extracts were prepared and analyzed by western blotting (*top*) or for proteasome activities using Suc-LLVY-AMC (*bottom*). (**b**) Downregulation of iRhom1 reduces proteasome activity and increases the accumulation of ubiquitin-conjugates. HEK293T cells were transfected with pSuper-Neo (Ctrl) or iRhom1-shRNA (sh-iRhom1) for 48 h and cell extracts were examined for chymotrypsin (Suc-LLVY-AMC), trypsin (Bz-VGR-AMC), and caspase (Ac-GPLD-AMC)-like activities. Bars represent mean values ± S.D. (*n* > 3). *P < 0.05. **(c**) iRhom1 modulates the accumulation of ubiquitin-conjugates. After transfection of HEK293T cells with either pSuper-Neo (sh-iRhom1 –) or iRhom1-shRNA (sh-iRhom1 +) for 48 h, cell extracts were analyzed by western blotting using an anti-ubiquitin antibody. (**d**) Overexpression effects of the Rhomboid protein family and their activity-dead mutants on proteasome activity. HEK293T cells were transfected with HA-RHBDL1, HA-RHBDL1 S312A, HA-RHBDL2, or HA-RHBDL2 S187G alone (*lower*), or together with GFP^U^ (*upper*) for 30 h. Cell lysates were analyzed by western blotting using the indicated antibodies (*upper*) or proteasome activity assay using LLVY-AMC (*lower*). Bars represent mean values ± S.D. (*n* > 3).

In a previous study, we employed a functional screening assay utilizing an unstable GFP–degron (GFP^U^) system to isolate novel regulators of proteasome activity^12^. After a gain-of-function screen using 6,200 cDNAs in mammalian expression vectors, we found several clones that greatly reduced the signal of GFP^U^ upon overexpression. Because iRhom1 was the most effective among them in reducing the GFP signal, we further analyzed the effect of iRhom1 on proteasome activity in detail. Ectopic expression of iRhom1 reduced the GFP^U^ fluorescence signal by 40% but did not affect the signal of cotransfected RFP (Figure S1a). Accordingly, western blot analysis revealed that ectopic expression of iRhom1 reduced the level of GFP^U^ protein in a concentration- and time-dependent manner (Fig. 1a and S1b). When we examined overexpression effects of more than ~100 cDNAs encoding ER membrane proteins, including amyloid precursor protein (APP), prolyl 4-hydroxylase subunit alpha-2(P4HA2), transmembrane emp24 protein transport domain containing 5 (CGI-100), and glucose-6-phosphatase (G6PT), on proteasome activity, we could not observe any significant change in our assay employing GFP^U^ (Figure S2a and b), indicating that elevation of proteasome activity by iRhom1 is not artificial result of overexpressing a polytopic membrane protein.

Measurement of proteasome catalytic activity using fluorogenic substrates in crude cell extracts revealed that depletion of iRhom1 expression in HEK293T cells significantly decreased the activities of three different enzymes of the proteasome and increased the accumulation of ubiquitin-conjugates (Fig. 1b,c). Conversely, overexpression of iRhom1 enhanced chymotrypsin-like activity and reduced the amount of ubiquitin conjugates (Figure S1c). Similar to the increase of enzyme activities in crude cell extracts, native gel analysis also revealed that overexpression of iRhom1 elevated enzymatic activities of both 30S and 26S proteasomes (Figure S2c) and reduced the accumulation of ubiquitin-conjugates induced by the proteasome inhibitor MG132 (Figure S2d). These results indicate that iRhom1 regulates proteasome activity. Because iRhom1 is a member of the Rhomboid protease family that regulates the EGF quality control system in the ER^16^, we evaluated whether other members of the Rhomboid family also affect proteasome activity. Overexpression and enzymatic assays revealed that RHBDL1 and RHBDL2 also reduced the level of GFP^U^ and elevated proteasome activity (Fig. 1d). In addition, the protease activity-dead mutants (RHBDL1 S312A and RHBDL2 S187G) also elevated proteasome activity as much as their wild-type did. These observations indicate that the Rhomboid protease family affects proteasome activity independently of its reported enzymatic activity.

### iRhom1 affects the assembly of proteasome complexes

**Figure 2:**
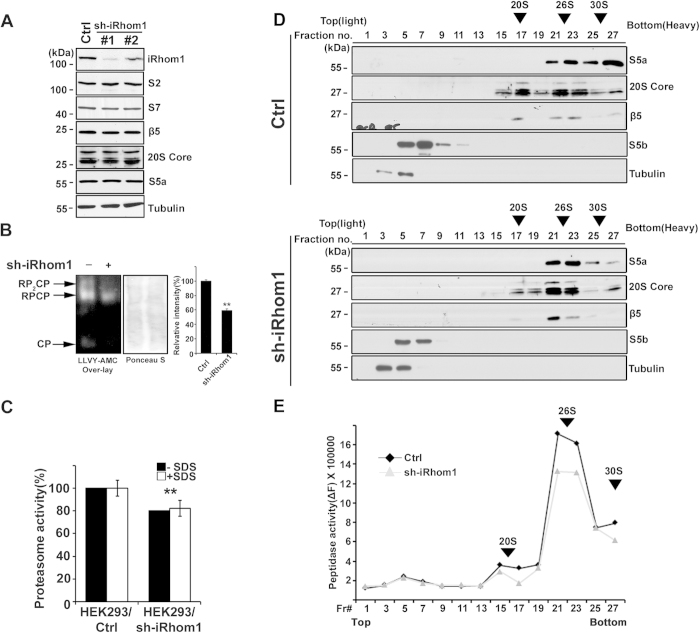
iRhom1 ensures the assembly of proteasome complexes. (**a**) Expression levels of proteasome subunits in iRhom1-knockdown cells. Cell extracts were prepared from control (Ctrl) and iRhom1-knockdown (sh-iRhom1 #1 and #2) HEK293 cells and analyzed by western blotting using the indicated antibodies. (**b** and **c**) Downregulation of iRhom1 impairs the assembly of proteasome complexes by native gel analysis. Cell extracts prepared from HEK293/pSuper-Neo (sh-iRhom1 –) or HEK293/iRhom1-shRNA (sh-iRhom1 +) cells were separated by native-PAGE and then analyzed by overlay assays using Suc-LLVY-AMC (b, *left*) or stained with Ponceau S (b, *middle*). RPCP, regulatory particle core particle; CP, core particle. The signals signal intensities of RP2CP and RPCP in figure (b) were quantified by densitometry and represented with bars for mean values ± S.D (*n* > 3) from at least three independent experiments (*right*). **P < 0.005. The same cell extracts were examined for proteasome activity in the presence or absence of 0.001% SDS. Bars represent mean values ± S.D. (*n* > 3). (c). (**d** and **e**) Knockdown of iRhom1 expression impairs the assembly of proteasome complexes in a fractionation assay. HEK293T cells were transfected with pSuper-Neo (Ctrl) or iRhom1-shRNA (sh-iRhom1) for 48 h and cell extracts were subjected to sedimentation analysis in a 10–40% (v/v) glycerol gradient. Fractions (250 μl) were collected and analyzed by western blotting after acetone precipitation (d) or proteasome activity assay using Suc-LLVY-AMC (e). The relative positions of 20S, 26S, and 30S proteasome complexes in the fractions are indicated with arrowheads.

To address how iRhom1 regulates proteasome activity, we established iRhom1-knockdown stable HEK293 cells using shRNA (Fig. 2a). Western blot and RT-PCR analysis revealed that iRhom1 deficiency did not affect the levels of the proteasome subunits, including S5a, 20S core, β5 and S2, that were examined (Fig. 2a and S3a). Native gel analysis followed by overlay assay using a fluorogenic enzyme substrate revealed that the enzymatic activities of 30S (RP_2_CP), 26S (RPCP), and 20S (CP) proteasomes were reduced in iRhom1 knockdown HEK293 cells (Fig. 2b). The reduced activities of proteasomes were not affected by the addition of SDS (Fig. 2c). These observations imply that assembly of the proteasomes might be regulated by iRhom1.

To resolve the steps of proteasome assembly in iRhom1-knockdown cells, we performed a fractionation assay using glycerol density gradient centrifugation and analyzed the fractions by western blotting using proteasome subunit-specific antibodies. Compared with control cells, iRhom1-knockdown led to a significant decrease in the levels of S5a, β5, and 20S core in three fraction regions: S5a in the first region comprising fractions 25–27 which contained the 30S proteasome, 20S core in the second region including fractions 17–19 which contained the 20S proteasome, and β5 in the third region comprising fractions 21–23 which contained the 26S proteasome (Fig. 2d). S5b was shown for control, which was not found in fully assembly proteasome complex^21^. Accordingly, an enzymatic assay revealed that the peptidase activity of the proteasome was also reduced by iRhom1-knockdown in the three fraction regions corresponding to the 20S, 26S, and 30S proteasome complexes, respectively (Fig. 2e). From similar fractionation assay using gel filtration^12^, we also observed reduction of 30S proteasome in iRhom1 knockdown cells and increase of assemble intermediates and free subunits (data not shown). Conversely, ectopic expression of iRhom1 did not affect the levels of proteasome subunits but increased the activities of the proteasome complexes as determined by glycerol density gradient fractionation analysis (Figure S3b and c, data not shown). These results indicate that iRhom1 regulates the activities of the proteasome complexes through their assembly.

### iRhom1 regulates microsomal proteasome activity in response to ER stress

**Figure 3:**
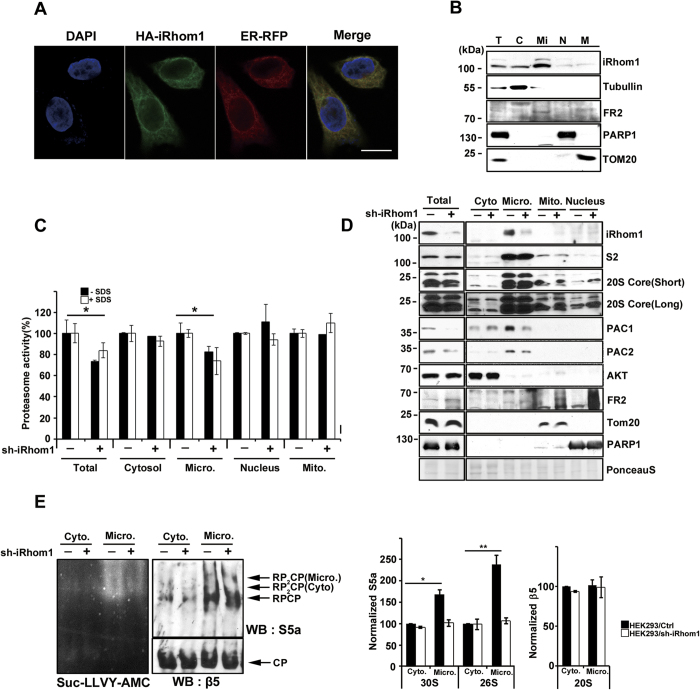
iRhom1 regulates ER-associated proteasome activity. (**a** and **b**) iRhom1 localizes in the ER of HeLa and HEK293T cells. HeLa cells were cotransfected with HA-iRhom1 and DsRed-Monomer-KDEL for 24 h, stained with anti-HA antibody (green) and Hoechst 33258 (DAPI) for nuclei, and then observed under a confocal microscope (a). The scale bar represents 20 μm. HEK293T cells were fractionated into the nuclear (N), mitochondrial (M), cytosolic (C), and ER-related microsomal (Mi) fractions by ultracentrifugation, and the fractions and total cell lysate (T) were analyzed by western blotting (b). (**c** and **d**) iRhom1-knockdown reduces proteasome activity in the microsomal fractions. Cell extracts prepared from HEK293/pSuper-Neo (sh-iRhom1 –) or HEK293/iRhom1-shRNA (sh-iRhom1 +) cells were separated into subcellular fractions as in (B), and then each fraction was examined for proteasome activity using Suc-LLVY-AMC in the presence or absence of 0.001% SDS (c) or analyzed by western blotting (d). The proteasome activities in total cell extracts and each fraction of control cells were considered 100. Bars represent mean values ± S.D. (*n* > 3). *P < 0.05. Short, short exposure; Long, long exposure. (**e**) Knockdown of iRhom1 **e**xpression reduces th**e** assembly and activity of microsomal proteasomes. The cytosolic and ER fractions were separated by native-PAGE and subjected to overlay assays using Suc-LLVY-AMC or western blot (WB) analysis using anti-β5 (WB: β5) and anti-S5a (WB: S5a) antibodies. The signals of S5a in 30S (RP2CP) and 26S (RPCP), and β5 in 20S (CP) on the blot was quantified by densitometry and represented with bars for mean values ± S.D. (*n* > 4). *P < 0.05, **P < 0.005 (*right*).

Because the proteasome is found in the nucleus and cytoplasm, and iRhom1 is known to be located in the ER, we evaluated the subcellular location in which iRhom1 affects proteasome activity. We first confirmed that HA-tagged iRhom1 colocalized with ER-RFP, an ER tracker, but not with Mito-RFP, a mitotracker, or LAPM2-GFP, a lysosome marker^22^, in the transfected HeLa cells (Fig. 3a and S4a). In the subcellular fractionation assay, we detected endogenous iRhom1 in the microsomal fraction containing ER (Fig. 3b). We then fractionated the cell extracts into the cytosol, nuclear, microsomal, and mitochondrial membrane fractions and compared the proteasome activities of the fractions prepared from wild-type and iRhom1-knockdown cells. Interestingly, catalytic activity of proteasome was reduced to 80% by iRhom1-knockdown only in the microsomal fractions (Fig. 3c). Please note that the level of PAC1, an assembly factor for the 20S proteasome, was significantly reduced in the microsomal fraction of iRhom1-knockdown cells, whereas there was no significant change in the amounts of proteasomal subunits S2 and 20S Core (Fig. 3d). Consistently, we found that the enzyme activities of the 26S and 30S proteasomes were reduced in the microsomal fraction of iRhom1-knockdown cells as determined by native gel analysis (Fig. 3e). Conversely, the overexpression of iRhom1 elevated proteasome activity in the microsomal fraction (Figure S4b and c).

**Figure 4:**
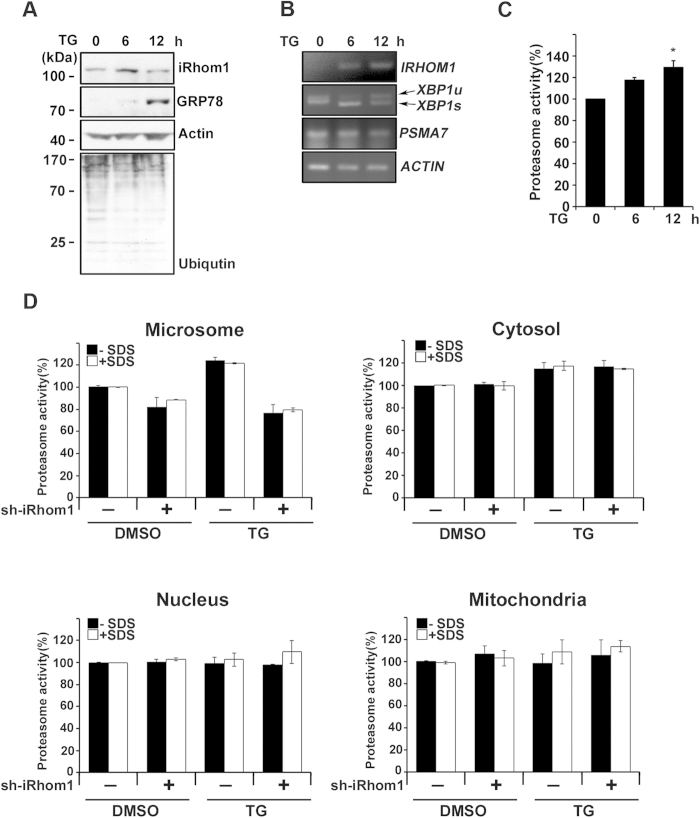
iRhom1 is increased by ER stress to enhance proteasome activity in the microsomal fraction. (**a**, **b,** and **c**) Thapsigargin treatment increases both iRhom1 expression and proteasome activity. HEK293T cells were treated with 2 μM thapsigargin (TG) for the indicated times and cell extracts were analyzed by western blotting (a) and for proteasome activity using Suc-LLVY-AMC (c). Total RNA was purified from those cells and analyzed by RT-PCR (b). *XBP1u*; unspliced XBP1, *XBP1s*; spliced XBP1. Bars represent mean values ± S.D. (*n* > 3). *P < 0.05. (**d**) Knockdown of iRhom1 expression impairs ER stress-induced activation of proteasomes in the microsomal fraction. After treatment with 2 μM thapsigargin (TG) for 6 h, cell extracts were prepared from HEK293/pSuper-Neo (sh-iRhom1 –) or HEK293/iRhom1-shRNA (sh-iRhom1 +) cells and fractionated into the nuclear, mitochondrial, cytosolic, and ER-containing microsomal fractions by ultracentrifugation and each fraction was examined for proteasome activity using Suc-LLVY-AMC in the presence or absence of 0.001% SDS. Bars represent mean values ± S.D. (*n* > 3).

We hypothesized that iRhom1 might regulate proteasome activity under ER stress. Therefore, we first examined the expressional regulation of iRhom1 under various ER stresses, such as treatment of cells with thapsigargin, A23187, and tunicamycin. We found that the level of iRhom1 protein was increased by thapsigargin at 6 h and then returned to the basal level at 12 h in HEK293T cells (Fig. 4a). Similarly, iRhom1 level was increased by ER stressors in other cells, such as Hep3B human hepatocarcinoma cells and SH-SY5Y neuroblastoma cells (Figure S5a). In addition, other inhibitors of protein synthesis, such as geneticin, puromycin, hygromycin, and cycloheximide, potently increased iRhom1 level in those cells. RT-PCR analysis revealed that iRhom1 mRNA level was gradually increased by thapsigargin at 6 h and remained high until 12 h in HEK293T cells (Fig. 4b) or by geneticin in SH-SY5Y cells (Figure S5b). Similarly, proteasome activity was increased by thapsigargin at 6 h and further increased at 12 h (Fig. 4c).

We then evaluated whether the increase in proteasome activity under ER stress is mediated by iRhom1. In contrast to the thapsigargin-treated control cells, the increase of proteasome activity in the microsomal fraction by ER stress was impaired by down-regulation of iRhom1; the proteasome activity of iRhom1-knockdown cells under ER stress was almost identical to that of untreated control cells (Fig. 4d). Similarly, native gel analysis revealed that the increase in enzyme activities of the 26S and 30S proteasomes in response to thapsigargin was impaired by iRhom1-knockdown in the microsomal fraction (Figure S5c). In addition, the puromycin-mediated increase in proteasome activity of SH-SY5Y cells was also impaired by iRhom1-knockdown (data not shown). These results suggest that iRhom1 plays an essential role in proteasome activation during ER stress.

### iRhom1 increases protein stability and dimerization of PAC1 and PAC2

**Figure 5:**
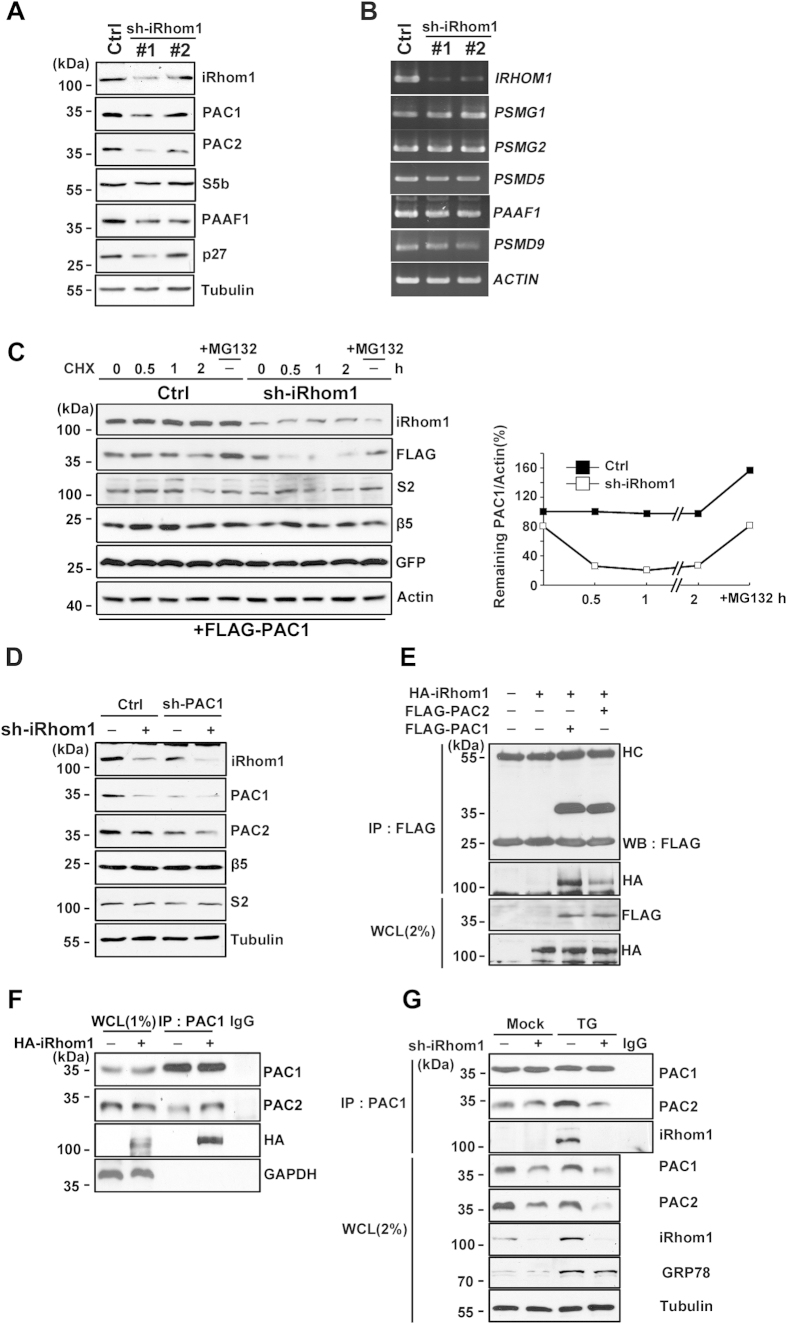
iRhom1 enhances the stability and dimerization of PAC1 and PAC2 proteins. (**a**) The amounts of PAC1 and PAC2 proteins are reduced by iRhom1-knockdown. Cell extracts prepared from HEK293/pSuper-Neo (Ctrl) or HEK293/iRhom1-shRNA (#1 and #2) cells were analyzed by western blotting. (**b**) iRhom1 does not affect RNA levels of proteasome assembly chaperones. Total RNA was isolated and analyzed by RT-PCR. (**c**) Downregulation of iRhom1 reduces the stability of exogenous FLAG-PAC1. SH-SY5Y/pSuper-Neo (Ctrl) or SH-SY5Y/sh-iRhom1 (sh-iRhom1) cells were cotransfected with FLAG-PAC1 and EGFP for 30 h and then incubated with 100 μg/ml cycloheximide (CHX) and/or 10 μM MG132 for the indicated times. Cell lysates were prepared and analyzed by western blotting (*left*). The signals of FLAG-PAC1 on the blots were quantified using ImageJ software (r*ight*). (**d**) Knockdown of PAC1 expression reduces the amount of PAC2 protein. HEK293/pSuper-Neo(sh-iRhom1 –) and HEK293/iRhom1-shRNA (sh-iRhom1 +) cells were transfected with pSuper-Neo (Ctrl), PAC1-shRNA (sh-PAC1), and/or iRhom1-shRNA for 72 h, as indicated, and cell lysates were analyzed by western blotting. (**e** and **f**) iRhom1 affects the interaction between PAC1 and PAC2. HEK293T cells were cotransfected with FLAG-PAC1, FLAG-PAC2, and HA-iRhom1 (e) or HA-iRhom1 only (f) for 30 h. Cell lysates were analyzed by immunoprecipitation (IP) assay with anti-FLAG-M2 beads or anti-PAC1 antibody, followed by western blotting using the indicated antibodies. HC indicate the heavy chains of immunoglobulin. (**g**) ER stress increases PAC1/PAC2 dimerization in an iRhom1-dependent manner. HEK293/pSuper-Neo (sh-iRhom1 –) or HEK293/sh-iRhom1 (sh-iRhom1 +) cells were left untreated (Mock) or incubated with 2 μM thapsigargin (TG) for 6 h. Then, cell extracts were prepared and subjected to immunoprecipitation (IP) assay using an anti-PAC1 antibody. Whole cell lysates (WCL) and the immunoprecipitates were analyzed by western blotting.

To gain insight into the role of iRhom1 in the regulation of proteasome assembly, we examined the expression levels of several proteasome assembly chaperones in iRhom1-knockdown cells. As seen in Fig. 3d, the level of PAC1 was significantly reduced in iRhom1-knockdown cells. We also found that the level of PAC2 as well as PAC1 was decreased in iRhom1-knockdown HEK293 cells (Fig. 5a). However, the levels of other assembly chaperones, such as S5b, PAAF1, and p27 proteins, were not related. In contrast to the protein levels, there was no difference in the levels of PAC1 and PAC2 mRNA (Fig. 5b). When we analyzed the stability of PAC1 protein in the presence of cycloheximide, we found that FLAG-PAC1 protein was stable for 2 h in SH-SY5Y cells. In contrast, FLAG-PAC1 protein was rapidly degraded within 30 min in iRhom1-knockdown SH-SY5Y cells, and this degradation was inhibited by the proteasome inhibitor MG132 (Fig. 5c). Similarly, we also found that PAC1 protein was rapidly degraded by iRhom1 knockdown in HEK293 cells (Figure S6b). Moreover, compared to HeLa cells, it appears that the half-life of PAC1 protein was increased in HEK 293 cells which showed higher levels of iRhom1 protein than HeLa cells (Figure S6a, b, and c). Together, these results suggest that iRhom1 regulates the stability of PAC1 and PAC2 proteins. Consistent with a previous report showing that the interaction between PAC1 and PAC2 increases their protein stability^23^, knockdown of PAC1 expression reduced the level of PAC2 protein (Fig. 5d). In addition, it appears that downregulation of both iRhom1 and PAC1 further reduced the level of PAC2 in HEK293T cells. Conversely, overexpression of iRhom1 counteracted the PAC1-dependent reduction of PAC2 level (Figure S7a).

Because PAC1 is highly detected together with iRhom1 in the microsomal ER fraction (Fig. 3d)^24^ and the initiation of proteasome assembly may occur in the ER^25^, we examined the possibility that iRhom1 interacts with PACs. Interestingly, immunoprecipitation analysis revealed that HA-iRhom1 interacted with FLAG-PAC1 and FLAG-PAC2 in the transfected cells (Fig. 5e and f) and this interaction was enhanced by iRhom1 overexpression (Fig. 5f and S7b). Conversely, knockdown of iRhom1 expression reduced the interaction of endogenous PAC1 and PAC2 (Fig. 5g). More interestingly, treatment with thapsigargin increased the interaction between PAC1 and PAC2, and this increase was impaired by iRhom1-knockdown (Fig. 5g and S7c). We also observed that iRhom formed a protein complex with PAC1 and PAC2 in the thapsigargin-treated cells (Fig. 5g and S6c). These observations indicate that iRhom1 regulates the interaction between PAC1 and PAC2

### iRhom1 relieves mutant Huntingtin aggregation in cells and Drosophila

**Figure 6:**
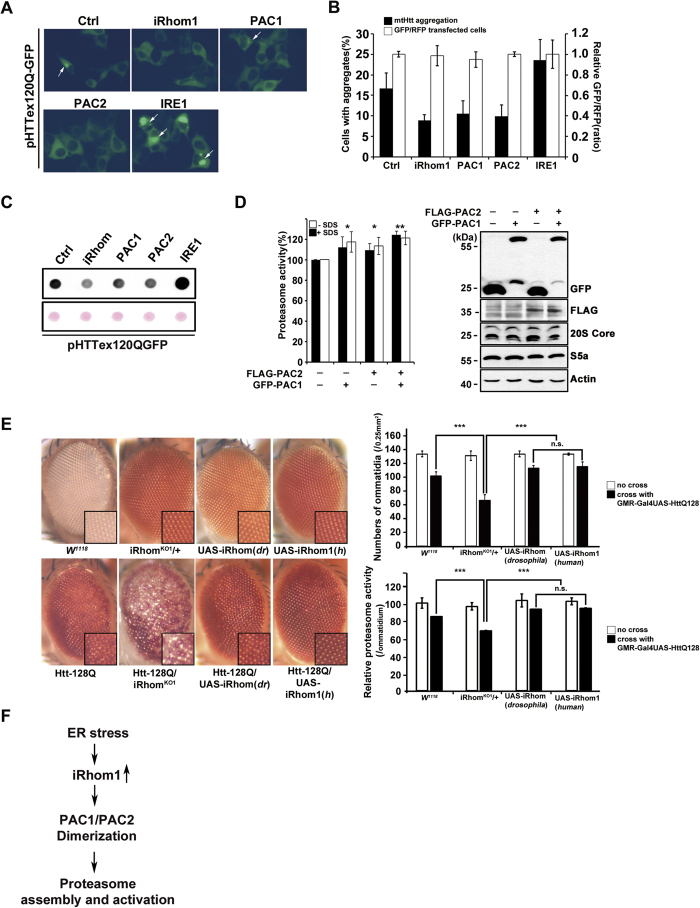
Expression level of iRhom1 modulates the aggregation of mutant Huntingtin in cells and the rough-eye phenotype in a fly model expressing Htt120Q. (**a**, **b,** and **c**) Ectopic expression of iRhom1 reduces the aggregation of mutant huntingtin (mtHTT) in HEK293T cells and SH-SY5Y neuroblastoma cells. HEK293T cells (a) and SH-SY5Y cells (b) were co-transfected with pHTTex120Q-GFP (mtHTT), RFP, and pcDNA (Ctrl), iRhom1, PAC1, PAC2, or IRE1. After 30 h, cells were examined for the aggregation (arrows) of mtHTT by fluorescence microscopy (a) and for the percentages of cells showing mtHTT aggregates among total GFP-positive cells (b). Transfection efficiency was normalized by RFP. Bars represent mean values ± S.D. (*n* = 3). HEK293T cell extracts were then prepared and subjected to a filter trap assay as described in Materials and Methods (c). (**d**) Ectopic expression of PAC1 and PAC2 elevates proteasome activity. HEK293T cells were transfected with PAC1, PAC2, or both PAC1 and PAC2 for 30 h. Cell extracts were prepared and analyzed for proteasome activity using Suc-LLVY-AMC (*left*) or western blotting (*right*). (**e**) iRhom is critical for the regulation of rough-eye phenotype and proteasome activity in a fly model expressing mutant huntingtin (Htt128Q). Wild-type (*w*^*1118*^), iRhom-knockout (KO1), iRhom (*Drosophila* form)-overexpressing [UAS-iRhom (*dr*)] or iRhom1 (human form)-overexpressing [UAS-iRhom1 (*h*)] flies were crossed with flies expressing Htt128Q. (*left*) The presence or absence of the rough-eye phenotype was then evaluated using a stereo microscope and the numbers of ommatidium of each drosophila eye were counted. Proteasome activity in the same numbers of fly heads in each group was measured and normalized by the numbers of ommatidium. Data are means ± SEM (***P < 0.005, *n *= 15). (**f**) Schematic diagram showing the proposed role of iRhom1 in proteasome activation under ER stress. ER-stress increases iRhom1, which results in enhanced dimerization of PAC1 and PAC2 and elevates proteasome activity.

Because it is known that proteasome activity is highly associated with the aggregation of mutant Huntingtin (mtHtt)^26^, we addressed whether iRhom1 contributes to the clearance of mtHtt. Ectopic expression of HTTex120Q-GFP, a GFP-fused segment of HTT exon 1-containing expanded polyglutamine (*n *= 120)^27^, exhibited large and disperse punctate fluorescent patterns in HEK293T cells (Fig. 6a). Coexpression of HTTex120Q-GFP with iRhom1 remarkably reduced puncta formation of HTTex120Q-GFP in HEK293T cells (Fig. 6a) and SH-SY5Y cells (Fig. 6b). As with iRhom1, coexpression with PAC1 or PAC2 also reduced the number of HTTex120Q-GFP aggregates in the same cells. However, overexpression of IRE1 increased the size and number of mtHtt aggregates, as described in our previous study^28^. Moreover, the filter trap assay showed that ectopic expression of iRhom1, PAC1, or PAC2 reduced the amount of SDS-insoluble aggregates of HTTex120Q-GFP in HEK293T cells (Fig. 6c). Accordingly, we found that overexpression of PAC1 and/or PAC2 significantly elevated proteasome activity (Fig. 6d). We previously confirmed that the HTTex120Q-GFP puncta were protein aggregates through immunostaining assays using an anti-ubiquitin antibody and detergent-resistance assays^29^. These results imply that iRhom1 reduces the accumulation of HTTex120Q-GFP in cultured cells.

To evaluate an *in vivo* role of iRhom1 in the clearance of mtHtt, we established iRhom-knockout (KO1), *Drosophila* iRhom [UAS-iRhom1 (*dr*)]- or human iRhom1 [UAS-iRhom1 (*h*)]-overexpressing flies, and crossed them with Htt-Q128 flies, which express Htt-Q128, display the rough-eye phenotype, and represent a Huntington disease model^30^. UAS-iRhom1 (*h*), UAS-iRhom (*dr*), and KO1 flies did not display any detectable alterations in eye phenotype (Fig. 6e, left upper). Consistent to the assays in cultured cells, overexpression of UAS-iRhom1 (*h*) or UAS-iRhom (*dr*) in Htt-Q128 flies relieved the rough-eye phenotype (Fig. 6e, left lower). Conversely, knockout of iRhom expression exacerbated the rough-eye phenotype in Htt-Q128 flies (Fig. 6e). Next, we measured proteasome activity in the head of drosophila. When we first examined developmental defect of drosophila eye in detail, we found that iRhom overexpression itself a little but significantly (5 ~ 10%) reduced the numbers of ommatidium in GMR-GAL4 line which expresses iRhom1 (*dr*) and iRhom (*h*) under GAL4 promoter. Then, measurement of the proteasome activity with same numbers of fly heads and normalization by the numbers of ommatidium revealed 5 ~ 15% differences of proteasome activity in iRhom-expressing/knockdown neurons compared to control fly (Figure S8a and b). Further, measurement of proteasome activity in the heads of iRhom/Htt-Q128 flies and normalization by the numbers of ommatidium also revealed that proteasome activity was significantly decreased by iRhom knockout in Htt-Q128 flies but increased by iRhom overexpression in Htt-Q128 flies (Fig. 6e, right). These results indicate that iRhom1 is effective mediator involved in the clearance of aggregation-prone protein, such as mtHtt.

## Discussion

Compared with other rhomboid-like family members, iRhom1 does not have a catalytic serine residue and inhibits the secretion of EGF, a rhomboid substrate, which is essential for cell survival^19^. While some growth factors, such as IGF-I, increase proteasome activity^31^, the proposed role of iRhom1 in the processing of EGF is inhibitory in growth factor secretion^15^. In addition, when we treated HEK293 cells with EGF, there was no significant difference in proteasome activity between wild-type cells and iRhom1-knockdown cells (data not shown). These observations all indicate that the stimulatory activity of iRhom1 on proteasome is not associated with the protease activity of the rhomboid-like family of proteins and is independent of EGF activity. Recently, several reports showed that rhomboid family Derlin-1 and RHBDL4 also facilitates ERAD and interacts with p97/VCP for substrate degradation independently of protease activity^32^,^33^. Perhaps, this interaction between rhomboid family and common factor p97/VCP may explain our observation that why all of rhomboid-like family members affects proteasome activity.

While it is not clear how microsomal proteasome activity is regulated by iRhom1, it is interesting to note that several assembly factors are detected in the microsomal fraction containing ER. In particular, large amounts of PAC1 and proteasomal subunits are consistently detected in the microsomal fraction. Moreover, a study in yeast showed that knockout of Pba2, a yeast homolog of PAC2, increases the accumulation and aggregation of misfolded proteins in the ER^34^, consistent to our results. Accordingly, it has been reported that chymotrypsin-like activity is more critical for cell as functions in ER-associated cellular processes and affects sensitivity to stress-induced degradation of misfolded protein^35^. We do not understand how overexpression of iRhom1 elevates only chymotrypsin-like activity, iRhom1, as an ER-resident protein, may regulate proteasome assembly through PACs in the ER. This idea is in a line with a report showing that the ER may be the subcellular organ in which proteasome assembly and activity are regulated^25^. From the fractionation assays following the protocol presented previously^36^,^37^, we see high levels of proteasome activity in the microsomal fraction which contains ER and nuclear membranes. While it is known that proteasomes, in general, are localized largely in the cytosol and nucleus^3^, more quantification for the proteasome activity in the ER fraction using diverse assays is needed.

It has been reported that *de novo* proteasome assembly can endure or overcome stressful conditions, such as oxidative stress, by increasing the transcription of proteasome subunits^38^ or assembly chaperones^39^. Similarly, it is reasonable to propose that proteasome assembly itself can be regulated to respond to stressful conditions through proteasome assembly factors, as previously shown by us in the case of S5b under chronic inflammation^12^. Like Rpn4 which regulates the transcription of yeast proteasome subunits has an extremely short half-life and responds to various stress signals^40^, we believe that PAC1 is regulated by iRhom1 to ensure the assembly of proteasomes under ER stress.

PAC1 and PAC2 form heterodimer and this dimerization increases their stability^23^. In our experiments, iRhom1 affected this heterodimerization and consequently the stability of PAC1 and PAC2 proteins. An important question that remains is, then, how iRhom1 regulates the stability of PAC1 and PAC2 proteins as well as their dimerization. HRD1, an E3 ubiquitin ligase in ERAD^41^, binds to PAC1, and this interaction is regulated by iRhom1 in our assays (data not shown). Moreover, ectopic expression of HRD1 or a dominant-negative p97/VCP mutant potentiated the increase of proteasome activity by iRhom1 (data not shown). Considering that PAC1 and PAC2 are actively involved in the assembly of the 20S proteasome complex^42^, iRhom1 may regulate the stability of PAC1 and PAC2 proteins probably through HRD1.

ER stress is induced by diverse cellular stressors, including calcium overload, glucose deprivation, or malformed proteins such as non-glycosylated or damaged proteins. Most abnormal secretory proteins are destined for degradation by ERAD, thereby reducing the load on cellular homeostasis caused by ER stress^4^. From our experiments, we found that the proteasome activity in the ER-containing microsomal fraction is affected by ER stress. Then, how is the proteasome activity in the microsomal fraction selectively regulated under ER stress? As several unfolded protein response (UPR) and ERAD-related genes expression are induced to overcome the stress^43^, the induction of iRhom1 at early stage of ER stress can be interpreted to function as a kind of ER stress sensor to regulate ER-associated proteasome activity. It has been noted that the Ufd-Npl4-p97/VCP protein complexes, which transports ERAD substrates from the ER to the cytosol, binds to the proteasome and this binding may recruit the proteasome to ERAD components^44^. We also found that iRhom1 binds to p97/VCP as other rhomboid proteases do^32^; however, this binding affected neither proteasome assembly nor proteasome activity in our assays. It thus appears that the regulation of microsomal proteasome activity is not associated with ERAD machinery; rather, it is associated with PACs. On the other hand, proteasome activity might be affected by additional signal as well as iRhom under the prolonged ER stress^45^.

Consistent with the stimulatory role of iRhom1 in proteasome activation in cultured cells, iRhom1 is critical in the regulation of proteasome activity and in regulating the aggregation and neurotoxicity of mtHtt in *D. melanogaster*. Our results show a peculiar role of iRhom1 in the regulation of proteasome activity under ER stress. Because iRhom1 is a type of inspection protein for secretory proteins and a mediator in ER stress-associated proteasome activation, it is conceivable that this quality control system may be coupled with stress response. Considering that mtHtt also causes ER stress^46^, the novel role for iRhom1 in increasing the activity of proteasomes under ER stress warrants further attention and proteasome regulation via iRhom1 provides insight into protein quality control.

## Materials and Methods

### Cell culture and transfection

HEK293T (human embryonic kidney cell) and SH-SY5Y (human neuroblastoma) cells were obtained from the American Type Culture Collection (ATCC) and cultured in DMEM containing 10% fetal bovine serum and penicillin/streptomycin at 37 °C under 5% CO_2_ (v/v). The cells were transfected using LipofectAMINE reagent (Invitrogen) according to the manufacturer’s instructions.

### Generation of stable cell line

HEK293 and SH-SY5Y cells were transfected either pSuper-Neo or pSuper-Neo-sh-iRhom1 for 24 h and then maintained in selection medium containing 2 mg/ml of G418 (Invitrogen) for 2 weeks. To form stable cell clones, a single cell was further cultivated and the expression level of each cell was analyzed by RT-PCR and western blotting.

### Genome-wide functional screening

Functional screening was previously described (Shim *et al.*, 2012). Briefly, for the primary screening, HEK293T cells were culture on a 96-well plate for 24 h and cotransfected with GFP^U^ and each of 6,200 cDNAs in a mammalian expression vector for 30 h. Then, iRhom1 was isolated among the putative positive cDNA clones reducing GFP^U^ fluorescence under a fluorescence microscope (Olympus).

### Plasmid construction

Plasmid of pCI-FLAG-iRhom1 was kindly provided by Dr. L.Y. Li (University of Pittsburgh, USA) and subcloned into pcDNA-HA. The pcDNA-FLAG-PAC1 and pcDNA-FLAG-PAC2 were kindly provided by Dr. S. Murata (University of Tokyo, Japan) and subcloned into EGFP-N1. HA-RHBDL1, HA-RHBDL2, RHBDL1 S312A, and RHBDL2 S187G were kindly provided by Dr. B. Cohen (Research Corporation Technology, USA). To construct iRhom1, PAC1 and PAC2 shRNA, heteroduplex oligomers containing 5′-UTR or CDS (iRhom1: 5′-AGC TGG ACA TTC CCT CTG C-3′, 5′-TGC CAG GAA CCA TGA GTG A-3′; PAC1: 5′-CCA GAA GCT TGA AGG GTT T- 3′; PAC2: 5′-GCA TAA ATG CTG AAG TGT A-3′) were synthesized, annealed, and cloned into pSuper-Neo (OligoEngine).

### Antibodies and western blotting

The following antibodies were used for western blotting and immunoprecipitation assay: anti-iRhom1 (RHBDF1, Sigma-Aldrich), anti-green fluorescent protein (GFP), anti-tubulin, anti-actin, anti-GRP78, anti-Ub anti-Tom20, anti-Foxred2 and anti-PARP1 (Santa Cruz Biotechnology), anti-S4, anti-S2, anti-β5, anti-S5a, anti-PAC1, anti-PAC2 and anti-20S core (BIOMOL Int). Cells were lyzed by sonication with RIPA buffer (50 mM Tris pH 7.5, 150 mM NaCl, 1% NP-40, 1% sodium deoxycholate, 0.1% SDS) with protease inhibitor cocktail. The lysates were clarified by brief centrifugation, separated by SDS-PAGE, and transferred onto nitrocellulose membranes using a Bio-Rad semi-dry transfer unit (Bio-Rad). Blots were blocked with 5% (w/v) non-fat dry milk in TBS-T solution [25 mM Tris pH 7.5, 150 mM NaCl, and 0.05% (w/v) Tween-20]. Then, the blots were incubated with the indicated antibodies and visualized using the ECL system (GE Healthcare).

### Assays for proteasome activities

Cells were rinsed by PBS twice and then lyzed by sonication using rectic buffer (30 mM Tris pH 7.8, 5 mM MgCl2, 10 mM KCl, 0.5 mM DTT, 1 mM ATP) with 1 mM of phenylmethylsulphonyl fluoride (PMSF). The lysates were clarified by centrifugation and the activities were measured by use of fluorogenic substrates (Suc-LLVY-AMC, Bz-VGR-AMC, Ac-GPLD-AMC) (BIOMOL) and a fluorometer (EnVision® Multilabel Reader; PerkinElmer) with excitation at 380 nm and emission at 460 nm.

### Reverse transcriptase-PCR

Total RNA was prepared using TRI reagent (Molecular Research Center) and cDNA was synthesized by reverse transcriptase (RT; Invitrogen), followed by PCR amplification. PCR was performed for 15-20 cycles using following synthetic oligonucleotides sets: *IRHOM* (5′-ATG GTG GGA CGG CTC ACC-3′, 5′-TTT TGG TGC AGA TCG GCC-3′), *XBP-1* (5′-GAA CCA GGA GTT AAG ACA GC-3′, 5′-AGT CCA TAC CGC CAG AAT CC-3′), *PSMA7* (5′-ATG AGC TAC GAC CGC GCC-3′, 5′-TGA TGC TTT CTT TTG-3′), *PSMG1* (5′-ATG GCG GCC ACG TTC TTC G-3′, 5′-GGT AAC ATG TCG ACA TGT G-3′), *PSMG2* (5′-ATG TTC GTT CCC TGC GGG G-3′, 5′-ATC TAT TTC AGG AAT GCA C-3′), *PSMD5* (5′-AGA TGT TTG GAT GC-3′, 5′-TCA TTC GGC TCC TTC-3′), *PAAF1* (5′-GGG AGT CCT TGC AGA TTG-3′, 5′-TCA GAG GTC AGA AAG CTG-3′), *PSMD9* (5′-ATG TCC GAC GAG GAA GCG-3′, 5′-CAG TGA CTG GAA GTT CTG G-3′) *PSMD4* (5′-AGG AGG AGG CCC GGC-3′, 5′-TCA CTT CTT GTC TTC C-3′), *PSMB10* (5′-ATG CTG AAG CCA GCC CTG-3′, 5′-CTC CAC CTC CAT AGC CTG-3′), *ACTIN* (5′-GAG CTG CCT GAC GGC CAG G-3′, 5′-CAT CTG CTG GAA GGT GGA C-3′).

### Subcellular fractionation

Cells were resuspended in buffer (20 mM HEPES pH 7.5, 10 mM KCl, 1.5 mM MgCl_2_, 1 mM EDTA, 1 mM PMSF), sheared by passing the suspension 30 times through a 26-gauge needle, and then incubated on ice for 20 min in the presence of 250 mM sucrose. A portion of the samples was saved to check the expression levels of protein between the samples. The cell lysates were centrifuged at 1,000 g for 5 min at 4 °C (nucleus), and the supernatant was further centrifuged at 8,000 g for 20 min at 4 °C (mitochondria). The supernatant was once again centrifuged at 100,000 g for 3 h at 4 °C. The resulting pellet contained the microsomal fraction, whereas the supernatant contained cytosol. The pellet of each step was collected, resuspended in buffer, and then used for proteasome activity analysis or native gel analysis.

### Glycerol gradient analysis

The 10–40% glycerol gradient analysis was examined as previously described^47^. Cells were lyzed by sonication in lysis buffer (50 mM Tris pH 7.5, 100 mM NaCl, 0.1 mM EDTA, 2 mM ATP), after which the lysates were centrifuged at 12,000 rpm for 30 min at 4 °C. The cell lysates were fractionated by 10–40% (v/v) glycerol density gradient centrifugation (22 h, 100,000 g) and 0.25 ml fractions were collected for analysis.

### Immunoprecipitation assay

HEK293T cells were lyzed in a RIPA buffer containing protease inhibitor cocktail. After centrifugation, the supernatant was incubated with FLAG-M2 bead (Sigma Aldrich) at 4 °C for 6 h. For endogenous immunoprecipitation, HEK293 cells were harvested and then lyzed in a RIPA buffer containing protease inhibitor cocktail. The cells were centrifuged at 12,000 rpm for 20 min at 4 °C and the supernatant was incubated with anti-PAC1 antibody overnight at 4 °C and pulled down by Protein G Sepharose beads (GE Healthcare).

### Immunocytochemistry

HeLa cells were cultured on a coverslip coated with poly-L-lysine and then transfected with HA-iRhom1 for 24 h. Cells were fixed with 4% paraformaldehyde and permeabilized with 0.1% Triton X-100. After blocking with 3% FBS in PBS, cells were incubated with anti-HA antibody for 2 h. Samples were observed on a confocal laser scanning microscope (LSM510, Carl Zeiss, Inc.).

### Native gel analysis

Native gel analysis was performed as previously described^48^. Cells were lyzed in a rectic buffer and cell lysates were separated by polyacrylamide gel electrophoresis (PAGE) on 4% (w/v) polyacrylamide native-gel at 4 °C (100 V). The gel was overlaid with a buffer containing Suc-LLVY-AMC for 30 min and the fluorescence signal was visualized on a UV trans-illuminator.

### Filter trap assay

HEK293T cells cotransfected with HTTex120Q-GFP for 24 h and washed twice with PBS. Cells were then resuspended in PBS containing 1 mM PMSF, sonicated, and then added with PBS containing 1% SDS. Prepared samples were subjected to a filter trap assay using a 96-well dot blot apparatus (Bio-Rad Laboratories). The nitrocellulose membrane was incubated and rinsed with PBS containing 1% SDS, and then analyzed by western blotting using anti-GFP antibody.

### Drosophila genetics

All crossbreeding experiment and maintenance were carried out at 25 °C. Transgenic flies expressing Htt-Q128 (GMR-GAL4/UAS-Htt-Q128) were generously provided by Dr. J.T. Littleton (Massachusetts Institute of Technology, USA). UAS-iRhom and *iRhom* knockout strain were kindly provided by Dr. M. Freeman (University of Oxford, UK). Wild-type (W^1118^) and IRE1 hetero knockout mutant (CG4583f02170) strains were purchased from the Bloomington Drosophila Stock Center at Indiana University. Transgenic strains expressing human iRhom1 were generated by embryonic injection (Korea Advanced Institute of Science and Technology, Korea).

## Additional Information

**How to cite this article**: Lee, W.J. *et al.* iRhom1 regulates proteasome activity via PAC1/2 under ER stress. *Sci. Rep.* **5**, 11559; doi: 10.1038/srep11559 (2015).

## Supplementary Material

Supplementary Information
