# Supplementary material for: iRhom1 regulates proteasome activity via PAC1/2 under ER stress
Source: Sci Rep. 2015 Jun 25;5:11559. doi: 10.1038/srep11559 (PMC4479803; doi:10.1038/srep11559)
Supplement: Supplementary Information [file srep11559-s1.pdf]

iRhom1 regulates proteasome activity via PAC1/2 under ER stress

WonJae Lee[1], YoungDoo Kim[1], Jisu Park[1], SangMi Shim[1], Jieun Lee[1], Se-hoon Hong[1], Hye-Hyun Ahn[1], Huikyong Lee[1], and Yong-Keun Jung[1*]

[1]Global Research Laboratory, School of Biological Science, Seoul National University, 1 Gwanak-ro, Gwanak-gu, Seoul 151-747, Korea

[*]To whom correspondence should be addressed:

[1]School of Biological Science, Seoul National University, 1 Gwanak-ro, Gwanak-gu, Seoul 151-747, Korea.

Telephone: 82-2-880-4401; Fax: 82-2-873-7524; E-mail: ykjung@snu.ac.kr

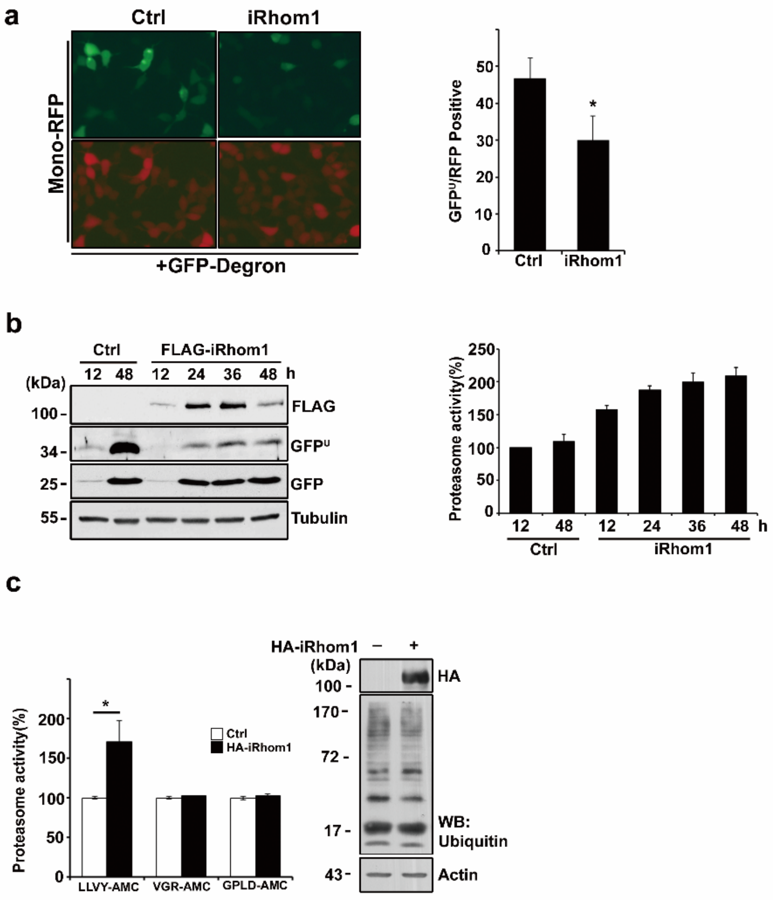

Figure S1. Stimulatory effect of iRhom1 overexpression on proteasome activity. (**a**) Accumulation of GFP^U-degron by iRhom1 overexpression. HEK293T cells were cotransfected with GFP^U, RFP, and either pcDNA3 (Ctrl) or iRhom1 for 36 h and then evaluated using a fluorescence microscope (*left*). Relative ratios of GFP^U-positive cells among RFP-positive cells (GFP^U/RFP) were determined (*right*). *$P < 0.05$. (**b** and **c**) Ectopic expression of iRhom1 increases proteasome activity. HEK293T cells were cotransfected with either pCI-FLAG (Ctrl) or FLAG-iRhom1 for the indicated times. Cell extracts were prepared and analyzed either by western blotting (*left*) or for proteasome activities using Suc-LLVY-AMC (*right*). (b). After transfection of HEK293T cells with either pcDNA-HA (HA-iRhom1 –) or HA-iRhom1(HA-iRhom1 +) for 30 h, cell extracts were analyzed for chymotrypsin (Suc-LLVY-AMC), trypsin (Bz-VGR-AMC), and caspase (Ac-GPLD-AMC)-like activities (*left*) or analyzed by western blotting using an anti-ubiquitin antibody (*right*) (c). Bars represent mean values ± S.D. ($n > 3$). *$P <$ 0.05.

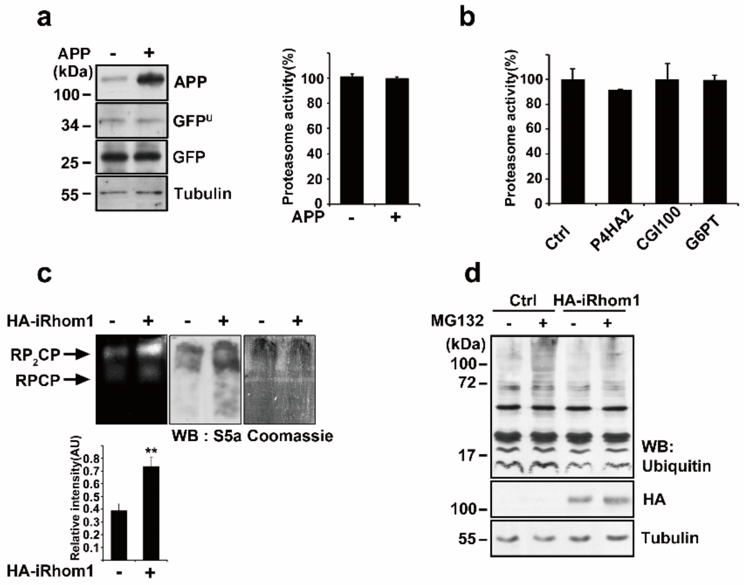

Figure S2. Effects of cDNAs encoding polytopic membrane proteins on proteasome activity and assembly and ectopic expression of iRhom1 increases proteasome assembly in native gel and reduces MG132 induced ub-conjugation. (**a**) APP overexpression does not affect GFP^U level and proteasome activity. HEK293T cells were cotransfected with either pcDNA3 (–) or APP (+) with GFP^U for 30 h. Cell extracts were prepared and analyzed either by western blotting (*left*) or for proteasome activities using Suc-LLVY-AMC (*right*). (**b**) Increase of ER membrane proteins does not affect proteasome activity. HEK293T cells were transfected with the indicated cDNAs for 30 h and cell extracts were then analyzed by Suc-LLVY-AMC for proteasome activity. (**c**) iRhom1 overexpression increases proteasome activity and reduces the accumulation of ubiquitin-conjugates. HEK293T cells were transfected with pcDNA-HA (HA-iRhom1 –) and HA-iRhom1 (HA-iRhom1 +) for 30 h and cell extracts were then separated by Native-PAGE and subjected to overlay assays using Suc-LLVY-AMC (*left*) or western blot (WB) analysis using anti-S5a antibody (*right*). The signal intensities of RP2CP and RPCP in figure (c) were quantified by densitometry and represented with bars for mean values ± S.D. ($n > 3$). $^{**}P < 0.005$ (*right*). (**d**) iRhom1 overexpression reduces the accumulation of ubiquitin-conjugates. HEK293T cells were transfected with pcDNA-HA (Ctrl) and HA-iRhom1 for 24 h and then incubated with or without 1 μM MG132 for 12 h. Cell extracts were then analyzed by western blotting.

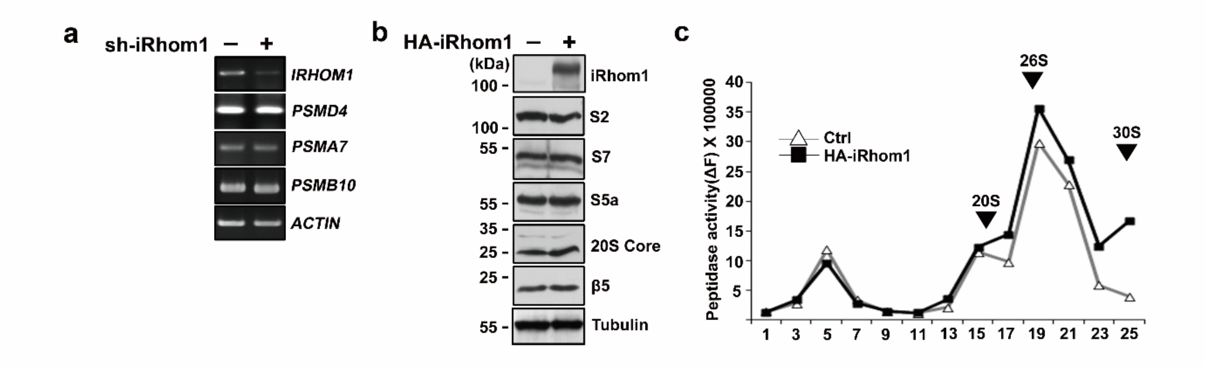

Figure S3. Effects of iRhom1 expression on proteasome assembly. (**a** and **b**) iRhom1 does not affect RNA or protein levels of proteasome subunits. Total RNA was purified from HEK293/pSuper-Neo (sh-iRhom1 –) or HEK293/sh-iRhom1 (sh-iRhom1 +) cells and analyzed by RT-PCR (a). HEK293T cells were transfected with either pcDNA-HA (HA-iRhom1 –) or HA-iRhom1 (HA-iRhom1 +) for 30 h and cell extracts were analyzed by western blotting (b). (**c**) Ectopic expression of iRhom1 increases proteasome activity in fractionation assays. HEK293T cells were transfected with pcDNA-HA (HA-iRhom1 –) or HA-iRhom1 (HA-iRhom1 +) for 30 h and cell extracts were fractionated by glycerol gradient centrifugation and the fractions were assayed for proteasome activity using Suc-LLVY-AMC. Arrowheads indicate the positions of proteasome complexes.

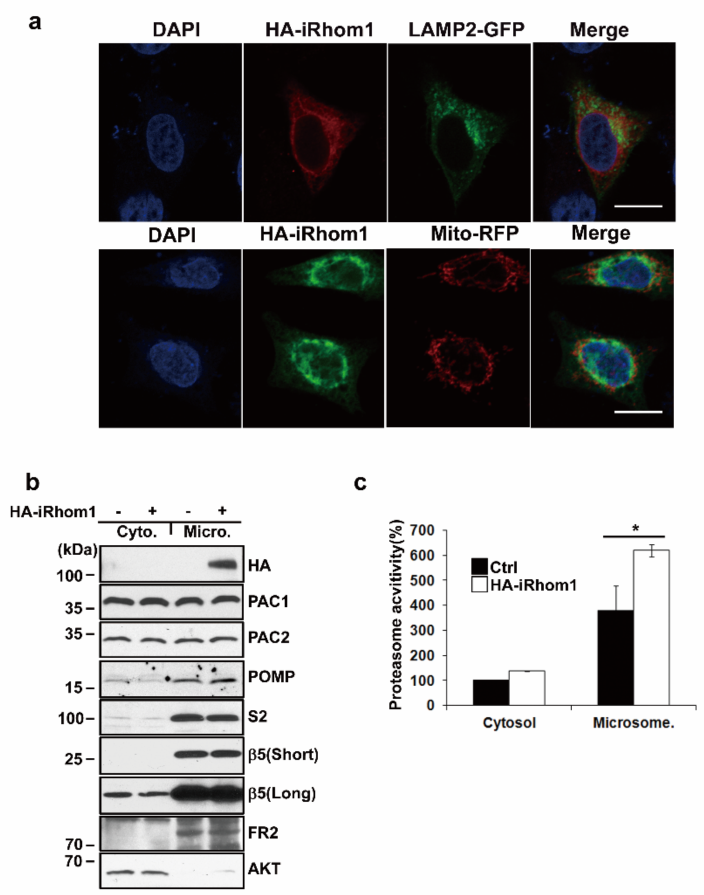

Figure S4. Ectopic expression of iRhom1 elevates proteasome activity in the microsomal fraction. (**a**) iRhom1 does not colocalize with LAMP2-GFP or Mito-RFP. After transfection of HeLa cells with HA-iRhom1 and either EGFP-LAMP2 or Mito-RFP for 24 h, cells were stained with an anti-HA antibody and Hoechst 33258 (DAPI) for nuclei and then evaluated by confocal microscopy. The scale bars represent 20 μm. (**b** and **c**) Overexpression of iRhom1 enhances proteasome activity in the microsomal fraction. After transfection of HEK293T cells with pcDNA-HA (HA-iRhom1 −) or HA-iRhom1 (HA-iRhom1 +) for 30 h, cell extracts were fractionated into the cytosol and microsome by ultracentrifugation. Each fraction was then analyzed by western blotting (b) and examined for proteasome catalytic activity using Suc-LLVY-AMC (c). Asterisks indicate non-specific bands. Bars represent mean values ± S.D. ($n > 3$). $^{*}P < 0.05$. Short, short exposure; Long, long exposure.

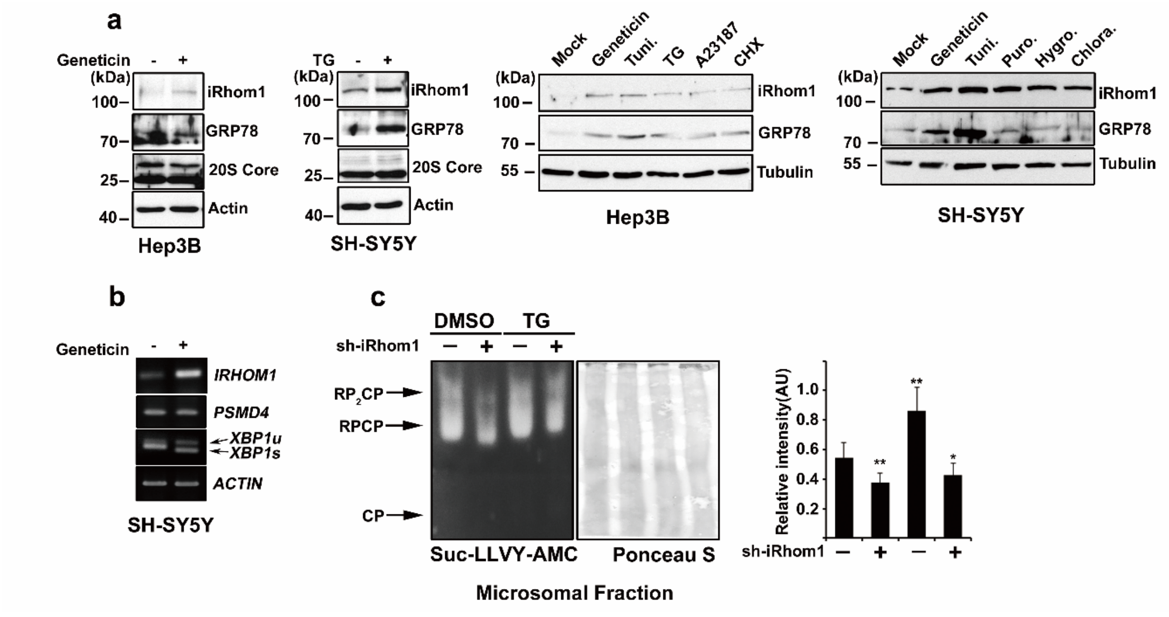

Figure S5. Increase in iRhom1 expression by stress signals. (**a** and **b**) Increase in iRhom1 by ER stressors and translation inhibitors. Hep3B cells and SH-SY5Y cells were left untreated (Mock) or incubated with geneticin (1 mg/ml) for 24 h (*left*) and 12 h (*right two*), tunicamycin (Tuni., 2 μM) for 24 h (*left*) and 12 h (*right two*), thapsigargin (TG, 1 μM), A23187 (1 μM), cycloheximide (CHX., 1 μg/ml), puromycin (Puro., 150 μM), hygromycin (Hygro., 150 μg/ml) and chloramphenicol (Chlora., 150 μg/ml) for 12 h (*right two*). Cell extracts were analyzed by western blotting (a) and total RNA isolated from SH-SY5Y cells was analyzed by RT-PCR (b). (**c**) Knockdown of iRhom1 expression impairs ER stress-induced proteasome assembly in the microsomal fraction. After treatment with 2 μM thapsigargin (TG) for 6h, cell extracts prepared from HEK293/pSuper-Neo (sh-iRhom1 –) or HEK293/iRhom1-shRNA (sh-iRhom1 +) cells were fractionated by ultracentrifuge. Collections of ER fraction were resolved by native-PAGE and then analyzed by overlay assays using Suc-LLVY-AMC (*left*). The blot was stained with Ponceau S (*middle*). The signal intensities of RP2CP and RPCP in figure (c) were quantified by densitometry and represented with bars for mean values ± S.D. (n = 5). *P < 0.05, **P < 0.005 (*right*).

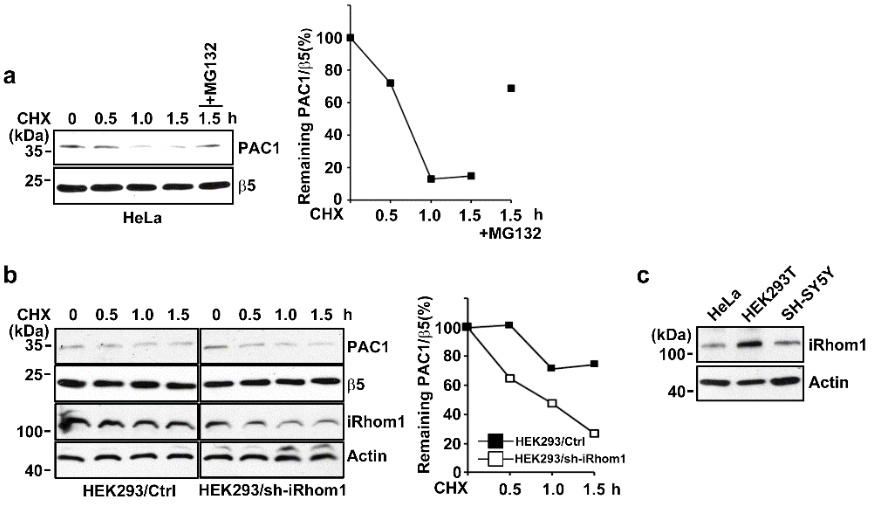

Figure S6. Downregulation of iRhom1 reduces the stability of PAC1 protein. (**a**) HeLa cells were treated with 200 μg/ml cycloheximide (CHX) with/without 10 μM MG132 for the indicated times and analyzed with western blotting (*left*). The signals of PAC1 on the blots were quantified using ImageJ software (*right*). (**b**) HEK293/pSuper-Neo (HEK293/Ctrl) or HEK293/sh-iRhom1 cells were incubated with 200 μg/ml cycloheximide (CHX) for the indicated times. Cell lysates were prepared and analyzed by western blotting (*left*). The signals of PAC1 on the blots were quantified as in (a) (*right*). (**c**) Total cell lysates of the indicated cell lines were analyzed by western blotting.

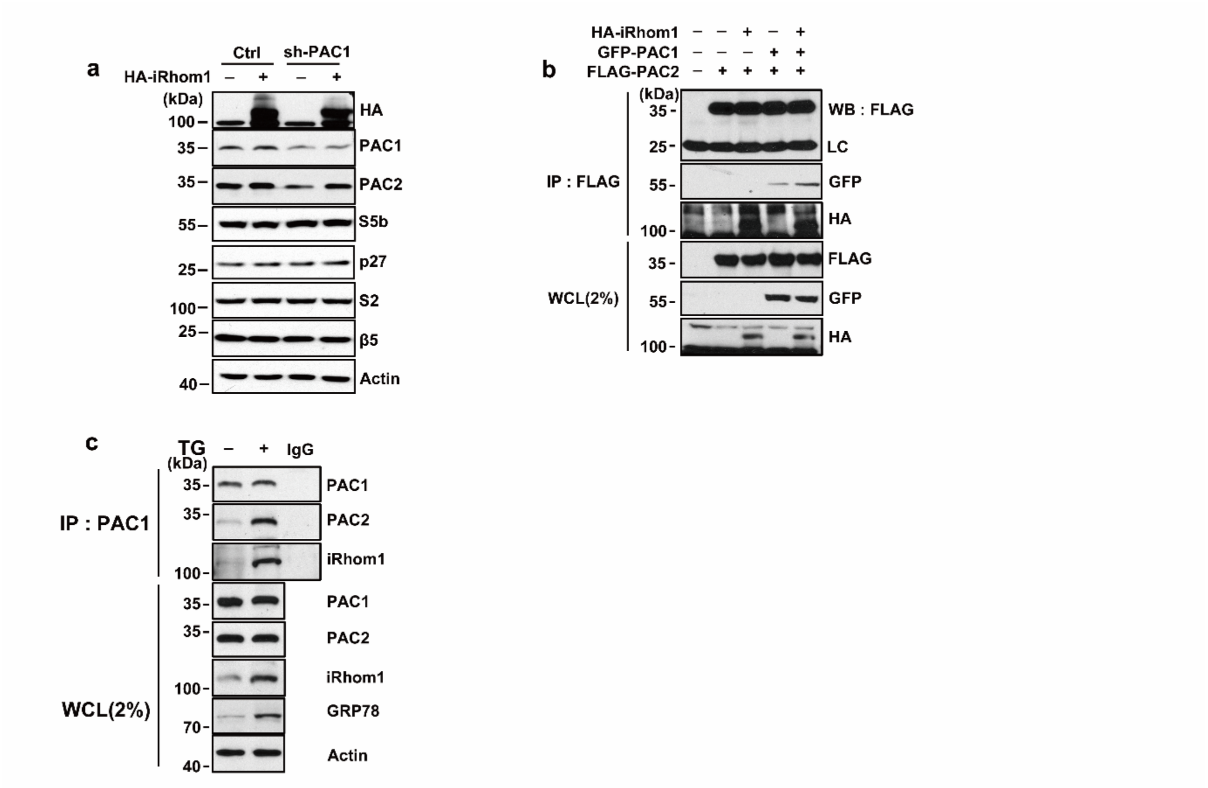

Figure S7. ER stress increases the interaction between PAC1 and PAC2. (**a**) Stability of PAC1 and PAC2 proteins are increased by iRhom1 overexpression. HEK293T cells were transfected with HA-iRhom1 and pSuper-Neo (Ctrl) or pSuper-Neo-PAC1 (sh-PAC1) for 70 h. Cell lysates were prepared and analyzed by western blotting. (**b**) iRhom1 affects the interaction of PAC1 and PAC2. HEK293T cells were cotransfected with GFP-PAC1, FLAG-PAC2, and HA-iRhom1 for 30 h. Cell lysates were analyzed by immunoprecipitation (IP) assay with anti-FLAG-M2 beads, followed by western blotting using the indicated antibodies. LC indicates the light chains of immunoglobulin. (**c**) Thapsigargin treatment increases PAC1 and PAC2 dimerization. HEK293T cells were treated with 2 μM thapsigargin (TG) for 6 h and cell extracts were analyzed by immunoprecipitation (IP) assay using an anti-PAC1 antibody. Whole cell lysates (WCL, 2% of input) and the immunoprecipitates were analyzed by western blotting.

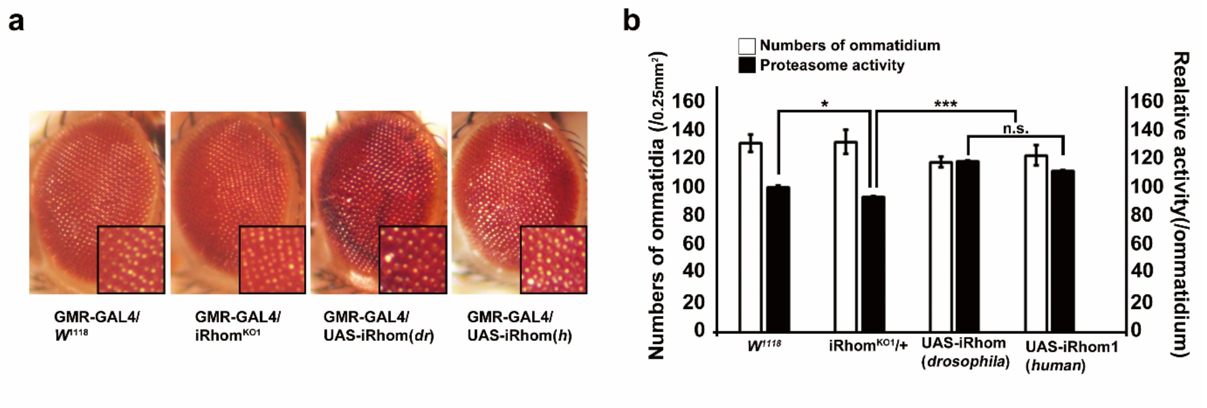

Figure S8. Overexpression of drosophila iRhom or human iRhom1 in drosophila eye shows mild disturbance in eye development and increases proteasome activity. (**a**) Single overexpression of drosophila or human form of iRhom leads to decrease in the numbers of ommatidium in drosophila eye. Wild-type (w^{1118}), iRhom-knockout (KO1), iRhom (*Drosophila* form)-overexpressing [UAS-iRhom (*dr*)] or iRhom1 (human form)-overexpressing [UAS-iRhom1 (*h*)]. (**b**) Overexpression of drosophila or human form of iRhom increases proteasome activity in the ommatidium of flies. Proteasome activity was measured in the fly head extracts and normalized by the numbers of ommatidium in each group. Data are the means ± SEM (****P* < 0.005, **P* < 0.05, *n* = 15).
